# Obesity: A Doorway to a Molecular Path Leading to Infertility

**DOI:** 10.7759/cureus.30770

**Published:** 2022-10-27

**Authors:** Rahnuma Ahmad, Mainul Haque

**Affiliations:** 1 Physiology, Department of Physiology, Medical College for Women and Hospital, Dhaka, BGD; 2 Pharmacology and Therapeutics, National Defence University of Malaysia, Kuala Lumpur, MYS

**Keywords:** sperm parameter, ovarian dysfunction, cytokines, adipokine, insulin resistance, inflammation, hypothalamic-pituitary-gonadal axis, infertility, obesity

## Abstract

The dramatic rise in obesity has recently made it a global health issue. About 1.9 billion were overweight, and 650 million global populations were obese in 2016. Obese women suffer longer conception time, lowered fertility rates, and greater rates of miscarriage. Obesity alters hormones such as adiponectin and leptin, affecting all levels within the hypothalamic-pituitary-gonadal axis. Advanced glycation end products (AGEs) and monocyte chemotactic protein-1 (MCP-1) are inflammatory cytokines that may play an important role in the pathophysiology of ovarian dysfunction in obesity. In obese males, there are altered sperm parameters, reduced testosterone, increased estradiol, hypogonadism, and epigenetic modifications transmitted to offspring. The focus of this article is on the possible adverse effects on reproductive health resulting from obesity and sheds light on different molecular pathways linking obesity with infertility in both female and male subjects. Electronic databases such as Google Scholar, Embase, Science Direct, PubMed, and Google Search Engine were utilized to find obesity and infertility-related papers. The search strategy is detailed in the method section. Even though multiple research work has shown that obesity impacts fertility in both male and female negatively, it is significant to perform extensive research on the molecular mechanisms that link obesity to infertility. This is to find therapeutics that may be developed aiming at these mechanisms to manage and prevent the negative effects of obesity on the reproductive system.

## Introduction and background

Excessive or abnormal fat accumulation leads to obesity and increases health risks. As per the World Health Organization (WHO), a person with a body mass index (BMI; normal range is 18.5 to 24.9) equal to or more than 25kg/m^2^ is considered overweight. A person is considered obese when their BMI is equal to or more than 30kg/m^2^, while someone with a BMI either equal to or more than 40kg/m^2 ^is morbidly obese [1]. There has been a dramatic rise in obesity prevalence globally, and it is now a worldwide health problem [2]. Among the adult global population in 2016, according to the WHO, about 650 million were obese, and about 900 million were overweight. WHO reported in 2021, among adults ≥18 years, globally 13% suffered from obesity (15% of them being female and 11% were male); overweight adults accounted for 13% of the worldwide population (40% of them were female and 39% were male individuals). The rate of obesity tripled by 2016 from 1975; additionally, this pandemic has exaggerated the issue [1]. A lack of balance between daily energy intake and expenditure results in excess weight gain. Multiple genetic, societal, and cultural factors contribute to obesity. Several genes are responsible for increasing weight and adiposity. Reduced physical activity, endocrine system disorders, insomnia, medications, intake of high sugar-containing food and excess carbohydrate, and reduced energy metabolism also result in obesity [3].

An increased risk of premature mortality was observed in obese subjects [4]. There is also a rise in comorbidity risk that includes type 2 diabetes mellitus (T2DM), cardiovascular disease, non-alcoholic fatty liver disease (NAFLD), asthma, endocrine disorders, polycystic ovary syndrome (PCOS), hypertension, osteoarthritis, malignancy (prostate cancer), neurodegeneration and accelerated aging [5-7]. The quality of life and life expectancy is affected negatively by such comorbidities and may impact an individual’s sexual and reproductive health [8]. Obesity may lead to infertility in both males and females. Both males and females suffer reproductive system complications from obesity [9-12]. Obese females suffer from irregular menstruation, endometrial thickness, and PCOS [13]. 

In males, obesity negatively impacts spermatogenesis and the quality of sperm such as sperm concentration, motility, viability, normal morphology, and sperm DNA fragmentation (SDF) [14]. Several studies have noted obese and overweight male subjects' higher prevalence of a trend of deteriorating semen quality. Dose-related relationship between increasing body mass index and subfecundity has been noted [14-16]. One meta-analysis observed that in comparison to normal-weight couples, there was a higher infertility risk among overweight couples. Obese male partners had statistically significantly higher infertility with an odds ratio (OR) of 1.66 (95% CI 1.53 to 1.79) than normal-weight sex partners [14]. Assisted reproductive technology success was also noted to be negatively influenced by male obesity [14]. In a study performed by Mushtaq et al., a significant association was found between raised male BMI equal to or more than 30kg/m^2 ^and reduced clinical pregnancy rate (OR: 0.78, 95% CI 0.63-0.98, p 0.03). This study also noted a significant association between raised male BMI equal to or more than 30kg/m^2^. It decreased live birth rates (OR 0.88, 95% CI 0.82-0.95, p 0.001) per in vitro fertilization (IVF) and intracytoplasmic sperm injection (ICSI) treatment cycle [17]. Currently, evidence suggests that not only female obesity but also male obesity may equally contribute to the quality of embryo and infertility pathogenesis [18].

Increased BMI in women is linked to a higher incidence of gynecological conditions like uterine fibroids and endometriosis [19,20], abnormal and excessive menstrual bleeding [21], PCOS [22], pregnancy complications like eclampsia and pre-eclampsia [23], infertility [24] and miscarriage [25]. PCOS patients often suffer from extra fat storage in the abdomen [26]. Consequently, obesity raises reproductive health disorders [27,28]. In obese women, androgen aromatization increases to form estrogen. Hyperandrogenemia results from hyperinsulinemia and resistance to insulin in obese women. There is the deterioration of hypothalamic-pituitary-gonadal axis regulation due to a decrease in growth hormone, sex hormone binding globulin and insulin-like growth factor binding protein, and an increase in leptin levels. Women with obesity have a lower rate of implantation and pregnancy [29]. Similarly, obesity in men leads to male infertility mediated through the altered hypothalamic-pituitary-gonadal axis, testicular steroidogenesis, metabolic dysregulation of insulin, cytokines, and adipokines, oxidative stress, and genetic and epigenetic changes [9,30,31].

This narrative review is based on research studies conducted among humans as well as animals to comprehend the link that exists between infertility and obesity. We have tried to highlight the pathophysiology involved in obesity and infertility among both sexes of humans. Finally, this paper attempts to point up obesity-related mechanisms that deteriorate human reproductive health and to uphold the quality of reproductive physiology. 

Material and methods

This is a review article that seeks to find the association between infertility and obesity along with the likely pathophysiology of infertility suffered by obese women and men. This review was done from April 2022 to July 2022. Electronic databases like Science Direct, Embase, Google Scholar, PubMed, and Google Search Engine were utilized using search words ‘Obesity,’ ‘Infertility,’ ‘Infertility in obese male individuals,’ ‘Infertility in obese female individuals,’ ‘Reproductive health and obesity,’ ‘Molecular mechanism for obesity and infertility’ to obtain related research works. Research works that were dated prior to the year 2000 and pieces of literature that could not be found in the English language were excluded from the study. 

Article highlights

I. Excessive or abnormal fat accumulation leads to obesity and gives rise to increased health risks. II. Both males and females may face complications in the reproductive system due to obesity. III. In obese females, reproductive health is negatively impacted through hypothalamic-pituitary-ovarian axis alteration and also similarly negatively affects the hypothalamic-pituitary-gonadal axis in males. IV. Hyperinsulinemia may increase androgen formation, which is aromatized into estrogen by adipose tissue, and negatively affect the hypothalamic-pituitary-ovarian axis. V. Raised advanced glycation end products (AGEs) levels (resulting from chronic inflammation in obesity) in follicular fluid were negatively associated with IVF outcomes, the number of retrieved and fertilized oocytes, the rate of pregnancy, and the negative impact on the quality of embryos. VI. Molecular mechanisms via which male reproduction is impaired due to obesity include hypogonadism with its influence on spermatogenesis, oxidative stress, adipokine formation, and inflammation. VII. Inflammation in the microenvironment and adipocyte dysregulation affects insulin signaling, which deteriorates testicular functions. VIII. The epigenetic modifications in obesity include alteration of histone, methylation of DNA, and change in miRNA, which may be transmitted to the offspring. IX. Each of the involved molecules and the possible pathways provide a potential target for therapeutics so that the reproductive health of obese individuals may be improved.

## Review

Obesity and female fertility

In females, reproductive health is affected negatively, possibly through hypothalamic-pituitary-ovarian axis alteration. Insulin levels in circulation are often high in obese females, which may lead to increased androgen formation by the ovaries [32]. The large quantity of adipose tissue then aromatizes the androgen to estrogen, exerting negative feedback on the hypothalamic-pituitary-ovarian axis. This may lead to an alteration in gonadotropin production [33]. Dysfunction of ovulation and abnormalities of menstruation result from such hormonal changes [12]. PCOS (manifested through hyperandrogenism and oligomenorrhea) is aggravated by hyperinsulinemia. Obesity contributes to insulin resistance, which may exacerbate PCOS features [34]. A vicious cycle ensues as androgen levels rise in polycystic ovarian disease, which promotes visceral fat deposition that causes hyperinsulinemia and insulin resistance, aggravating the production of adrenal and ovarian androgen [35].

Obese female subjects have been observed to take a longer time to conceive. Cohort studies on Danish female individuals who were planning to conceive noted that a rise in BMI was associated with a fall in fecundability ratios [36,37]. Subfertility has been observed in obese females despite normal ovulatory function. A study by van der Steeg et al. on 3000 Dutch women having regular menstrual cycles noted that BMI >29 kg/m^2^ was related to a linear fall in conception probability [38]. Gesink et al. carried out a cohort study on over 7000 female subjects in America and found a lowering of fecundity in an obese female with a regular menstrual cycle [39].

**Figure 1:**
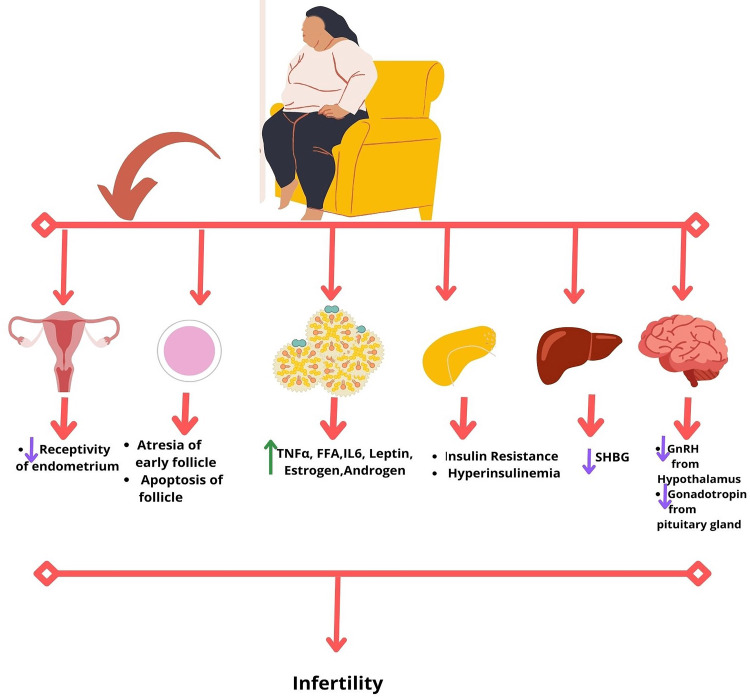
The complications of obesity in females leading to infertility, including early follicle atresia, follicle apoptosis, reduced receptivity of endometrium, release of inflammatory cytokines, adipokines like leptin, increased androgen and estrogen formation due to adiposity; hyperinsulinemia and insulin resistance; reduced sex hormone binding globulin from the liver; reduced gonadotropin releasing hormone from hypothalamus and decreased gonadotropins from pituitary gland. SHBG: sex hormone binding globulin. GnRH: gonadotropin-releasing hormone. TNFα: tumor necrosis factor alpha. FFA: free fatty acids. IL6: interleukin 6.  ↓: Decrease. ↑: Increase. Image Credit: Rahnuma Ahmad

Assisted reproductive technology (ART) results are also affected by obesity. Smaller oocytes, with less possibility of being normally fertilized, have been observed in obese female subjects receiving IVF [12,40]. Several studies have reported an association between rising BMI and a negative effect on live birth rates [41-45]. A study with obese and overweight women undergoing ART noted a moderate impact on live birth rates (OR 0.90) [46]. A retrospective cohort study done by Xue et al. on women undergoing ART noted that in subjects with BMI >= 24 to <28, the cumulative live birth rate was reduced significantly (OR 0.82, 95%CI 0.74-0.89, p <0.0001) when compared to women with normal weight. Also, in the study, the cumulative live birth rate in subjects with BMI >= 28 was found to be significantly decreased (OR 0.60, 95% CI 0.51-0.70, p <0.0001) when compared with the women with normal weight [43]. Another systemic review done by Koning et al. found the pooled ORs for overweight versus normal weight women on live following ART to be (OR 0.90, 95%CI 0.82-1.0) and concluded that raised BMI marginally decreases the success rate of pregnancy following ART [46]. Figure 1 shows the possible complications arising in the female reproductive system due to obesity.

Obesity and the hypothalamic-pituitary-ovarian axis

The functioning of the hypothalamic-pituitary-ovarian axis is impacted by body fat in females through both central and peripheral mechanisms [47,48]. Previous studies have shown that premature puberty is linked to obesity, while BMI below normal may result in delayed puberty [49]. Such findings have led to more research into the pathways and mediators of metabolism which act on the hypothalamic-pituitary-ovarian axis and thus affect fertility [50].

The discovery of the actions of adipokines has led to the understanding that infertility pathophysiology involves adipose tissue dysfunction since it is imperative for the adipokines to be within normal levels for sustaining the hypothalamic-pituitary-gonadal axis and ovulation regulation processes. Such adipokines secreted by adipocytes include leptin, adiponectin, resistin, omentin, and visfatin. Abnormalities in the adipokines may also result in insulin resistance and T2DM [51]. Resistance to insulin then may lower the pulse amplitude of the luteinizing hormone as well as the mean release of luteinizing hormone in the pituitary gland in obesity which may cause luteal phase impairment [52-56]. In the following segments, we will discuss the role of some of the adipokines abnormalities on the hypothalamic-pituitary-ovarian axis.

Role of leptin on the hypothalamus-pituitary-ovarian axis

The influence of leptin on the hypothalamic-pituitary-ovarian axis has been studied widely. Leptin has been observed to be significant fertility as well as puberty gatekeeper via its gonadotropin-releasing hormone-pulsed stimulatory effect [57,58]. Leptin receptors are found in the gonadotropin releasing hormone (GnRH) secreting cells of the hypothalamus. Since GnRH is necessary for the release of gonadotropins, it is a vital determinant for hypothalamus-pituitary-ovarian axis stability [59,60]. Leptin is also involved in the central pathways which govern the release of follicle-stimulating hormone and luteinizing hormone [61]. Leptin levels in the periphery are directly associated with body fat amount, and an increase in body fat results in an increase in leptin secretion [62,63]. Obesity also causes the development of leptin resistance centrally owing to the downregulation of the expression of leptin receptors and thus may disrupt the hypothalamic-pituitary-ovarian axis [64,65]. Even though the brain may develop resistance to leptin, the other tissues, like ovaries, continue to be leptin sensitive and thus are affected by the high levels of leptin in the circulation in obese individuals [50].

Role of leptin in ovulatory dysfunction

White adipose tissue secretes leptin, and leptin levels in serum are associated positively with adipose tissue amount. High leptin levels in obese females suggest resistance to leptin in these individuals [56,66]. Leptin’s inhibitory effect has been suggested by studies, particularly in the early stages of the development of follicles [56]. Studies related to the outcome of IVF observed a negative correlation between leptin and female reproductive physiology. A study evaluating levels of serum leptin among IVF recipients observed an association between a higher ratio of leptin to BMI and good quality embryos were low in number; thereby, the rate of success of IVF and pregnancies was minimum [66-68]. Downregulation of the gene for the anti-Mullerian hormone pathway of Janus kinase/signal transducers and activators of transcription was noted when exposure of human cumulus and granulosa cells was done to leptin in vitro [69]. This suggests the possibility of dysfunction of ovulation. High levels of leptin hinder the development of follicles [70,71].

Reduced adiponectin level in obesity and infertility

Adiponectin is produced by adipose tissue and increases during starvation [72]. Adiponectin is inversely related to adiposity and increases sensitivity to insulin [73]. In some genetic polymorphisms, the adiponectin level is reduced, leading to insulin resistance, T2DM, and metabolic syndrome [74]. In the case of obese female subjects’ low adiponectin levels have been observed along with increased inflammatory cytokines like tumor necrosis factor alpha (TNF-α), C-reactive protein (CRP), and interleukin-6 (IL6) [56]. These inflammatory cytokines can cross the blood-brain barrier and cause inflammation in the hypothalamus [75,76]. This leads to the promotion of obesity and resistance to insulin [75].

Adiponectin, insulin-like growth factor 1 (IGF-1), and insulin act on granulosa cells in the follicular phase of the ovarian cycle. It possibly causes upregulation of the StAR (steroidogenic acute regulatory protein) gene as well as increases estradiol and progesterone production in the ovary via IGF-1, as observed in the ovaries of rats [56,77]. Adiponectin causes upregulation of cyclooxygenase-2 (COX-2) and vascular endothelial growth factor (VEGF) in the follicular phase of the ovarian cycle and thus causes vasodilation [78,79]. Ovarian dysfunction, including the high number of atretic follicles, lower number of oocytes, prolonged cycles, and reduced activity of luteinizing hormone (LH) receptors, has been noted in adiponectin knockout mice [80]. Women who conceived following IVF were noted to have higher adiponectin levels, and a positive correlation was found with the number of retrieved oocytes [81]. Adiponectin has receptors at all levels of the reproductive axis and acts by promoting sensitivity to insulin [56].

Role of resistin in female fertility, obesity, and insulin resistance

Resistin is adiponectin secreted by mononuclear cells of blood like macrophages and also by adipose tissue stromal cells. However, the mRNA of this adiponectin is also noted in the hypothalamus-pituitary axis [82,83]. The polymorphism of genes for resistin is related to the BMI of women with PCOS. A study observed that when overweight women with PCOS were treated with the insulin sensitizer rosiglitazone, serum resistin lowered significantly, suggesting that resistin plays a part in adiposity and insulin sensitivity [84].

Visfatin's role in female obesity and infertility

Visfatin is adiponectin produced by different cells and tissues, including lymphocytes, adipocytes, muscle, liver, fetal membrane, and bone marrow. Studies in vitro have noted that glucose uptake by muscle cells and adipocytes is stimulated by visfatin [82]. A meta-analysis found a significant rise in visfatin levels in obese and overweight individuals and subjects with metabolic syndrome and T2DM [85]. Expression of the visfatin gene has also been noted to rise in women suffering from PCOS when compared to women without PCOS [86].

Somatotropic axis in obese female

Growth hormone has a stimulatory effect on follicles and prevents them from becoming atretic. Growth hormone, along with gonadotropins, promotes the genesis of follicles in the later stages, the development of the follicle, which is dominant, and luteinization. Growth hormone also raises progesterone and estrogen formation and promotes changes in the uterus's myometrium and endometrium for successful reproduction [87].

A decrease in growth hormone production and a rise in the clearance of growth hormone have been noted in the case of obese individuals in different studies [88,89]. The possible pathophysiology behind the decrease in growth hormone in plasma includes GnRH, somatostatin, and ghrelin dysregulation. Also involved may be excess free fatty acid in circulation and hyperinsulinemia. A decrease in IGF binding protein (IGFBP)-1 and IGFBP-2 in obese individuals results in the rising of free IGF, which in turn may cause suppression of growth hormone release through a feedback mechanism [50]. Hyposomatotropism is therefore suggested to be a characteristic of obesity [90] which may alter functions of the ovary and endometrium mediated through growth hormone and thus have a negative impact on female fertility [50].

Effect of obesity on the endometrium

Studies done on diet-induced obese mice reported impairment of decidualization of the endometrium, suggesting obesity also targets the endometrium [91]. Human studies have also observed a fall in the decidualization of stroma in obese women [92]. The inflammatory cytokines, reactive oxygen species (ROS), and haptoglobin, a marker of inflammation found to rise in obese females with repeated miscarriages, may be responsible for this phenomenon [93,94]. Downregulation of ERK signal transduction (a part of MAPK/ERK pathways) required for endometrial trophoblast invasion has been noted in obese women [95]. Such mechanisms may lead to a decrease in implantation and a raised rate of miscarriage in obese women [50]. The receptivity of endometrium may also be reduced because of a decrease in glycodelin, IGFBP1, hyperestrogenemia, hyperinsulinemia, and leptin pathway dysregulation [96-98].

Insulin resistance, obesity, and infertility in women

Insulin resistance, along with obesity, harms reproduction [99,100]. Pancreatic β-cells of islets of Langerhans are stimulated by adipose tissue to cause the release of insulin [101]. Hyperinsulinemia causes a rise in steroid hormones like androgens and estrogen in circulation. Insulin causes up-regulation of CYP17A1 enzymes that increase the production of androgens in the ovary and adrenal gland [102]. Insulin promotes the activity of LH to raise the formation and release of androgen from the ovary [103]. There is an association between insulin resistance and a rise in leptin levels [104]. Increased levels of leptin in circulation result in resistance to leptin and therefore increase in resistance to insulin [56].

**Figure 2:**
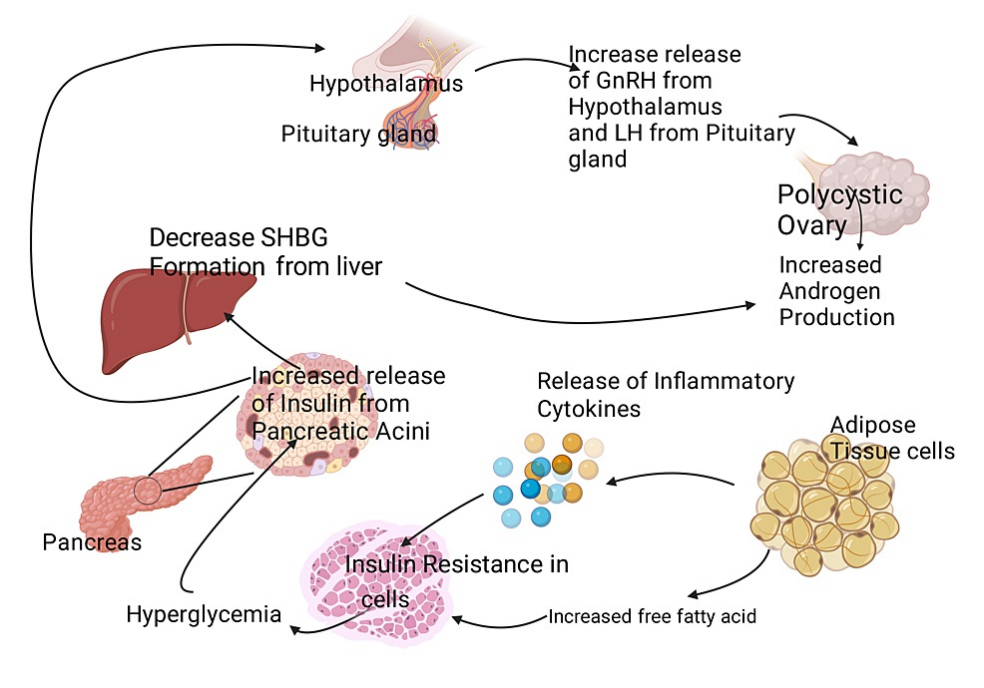
Free fatty acid due to adiposity leads to insulin resistance which in turn causes hyperinsulinemia. Raised insulin levels cause a decrease in SHBG and an increased level of GnRH and LH, which causes hyperandrogenism. SHBG: sex hormone binding globulin. GnRH: gonadotropin-releasing hormone. LH: luteinizing hormone. This figure has been developed using BioRender (https://biorender.com) License number: RU24HIWXUW. Image Credit: Rahnuma Ahmad.

Hyperinsulinemia is linked to higher levels of LH and hyperandrogenism [105] (Figure 2). Insulin modulates the pituitary gland’s receptor for GnRH and causes higher secretion of LH following stimulation by GnRH [106]. Insulin also increases the activity of follicle stimulating hormone (FSH) by promoting steroid synthesis in ovaries and increasing responsiveness to LH [105]. Severe insulin resistance has been linked to hyperandrogenism and enlargement of ovaries regardless of gonadotropin levels. Long-term elevated insulin levels may result in a rise in its receptor autophosphorylation. This, in turn, may cause the inactivation of GSK3 (a downstream transducer) and disrupt spindles in developing oocytes [107,108]. Disruption of chromatin remodeling during oocyte development and, thus lower oocyte quality has been noted in mice exposed to high insulin levels [107]. Despite resistance to insulin in the body's periphery, the pituitary gland is sensitive to insulin in mouse models [106].

The reproductive cycle in the experimental animal has been found to improve when insulin signaling is disrupted in case of obesity induced by diet. This indicates that pituitary dysregulation of LH in obesity may be mediated through insulin [106]. In the case of mice with knocked-out insulin receptors in theca cells, it was noted that the reproductive cycle improved, suggesting insult acts in coordination with the hypothalamic-pituitary-ovarian axis to disrupt the reproductive cycle [102]. Insulin is, therefore, a significant role player in reproduction in the case of females due to its effect on the hypothalamic-pituitary-ovarian axis and its connection to adiponectin and leptin [56,82,109].

Polycystic ovary syndrome and obesity

One of the most common reasons for infertility due to an anovulatory cycle is PCOS; about 50% of women with PCOS suffer from obesity [110,111]. Obesity in women with PCOS has frequently affected the menstrual cycle along with ovulation, pregnancy, and rate of live births negatively [112,113]. Studies have noted poor response to ovulation induction treatments and low recruitment of oocytes despite the use of a higher concentration of gonadotropin at the time of assisted reproductive technology application in obese women with PCOS in comparison to those without obesity [114,115]. Although the pathological mechanisms that lie behind the effects of obesity on fertility in women with PCOS remain unclear, obesity-induced insulin resistance, hyperandrogenism, and hyposomatotropinism may be mechanisms at play [116-118]. Studies have observed that changes in dietary habit and ensuring standard physical activity improves reproductive health, including positive effects on ovulation and menstrual cycle as well as fertility in obese women with PCOS [119,120]. Such benefits were observed even when about 5-10% weight loss took place [121-123]. Prevalence of irregularities in menstruation lowered to 7.7% from 56.2%, and infertility prevalence decreased to 4.3% from 18.2% in obese women with PCOS about one year after they underwent bariatric surgery [124].

Obesity-induced inflammation with molecular change and female infertility

Obesity is a condition with chronic inflammation with macrophage infiltration in tissues. There is a direct correlation between adipose tissue infiltration by macrophages and the production of adipokines/chemokines like MCP-1 and adiposity degree [125,126]. There is a similarity between the pattern of macrophage infiltration in chronic inflammatory disorders like rheumatoid arthritis and that in the case of chronic inflammation in obesity [56,126]. Obese female subjects also have raised circulating MCP-1, AGE, and markers of inflammation like TNF-α, CRP, and IL-6 [127,128].

Diet-induced obesity may promote the formation of AGE which in turn cause the expression of the gene for the MCP-1 [56,129]. The high-reactive AGE molecule comprises cross-linked protein, nucleic acid, lipid, and glucose [130]. Several studies have been carried out on the effect of the AGE molecule on reproduction [131,132]. Transport of glucose in granulosa cells has been noted to be hampered by AGE molecule [131]. MCP-1 knocked out obese mice who did not have ovarian dysfunction, which suggests that the absence of MCP-1 can be protective against the ovarian dysfunction induced by obesity [133]. Raised levels of MCP-1 in serum were linked to negative outcomes in women who took part in IVF, especially in subjects with a lower reserve of ova [134]. Also, higher levels of AGE in the fluid of follicles were correlated negatively with the outcome of IVF, the number of retrieved and fertilized oocytes, the pregnancy rate, and the lower number of embryos [132]. Thus, such findings indicate inflammatory AGE/MCP-1 activation in obesity impacts the female gonads.

The outcome of assisted reproductive technology in obese patients

A rising BMI has been found in several studies to be correlated negatively with the rate of live birth, implantation, and pregnancy [135,136]. Live birth rates and pregnancy in obese women may be up to 50% lower than in non-obese women [135,137]. Even after being given a more significant dose of gonadotropins, obese women attained lower-ranking levels of estradiol in serum and retrieval of oocytes in lower numbers [135,138]. Obese women have smaller oocytes with a lower potential for fertilization, a decrease in blastocyst formation, and a lower number of trophectoderm cells [139]. There may be disruption of endometrium receptivity and poor implantation rate due to obesity, possibly resulting from inflammation of the endometrium [135,140]. Markers of inflammation like TNF-α and IL-6 have been related to a poorer rate of implantation [128].

Obesity and male infertility

**Figure 3:**
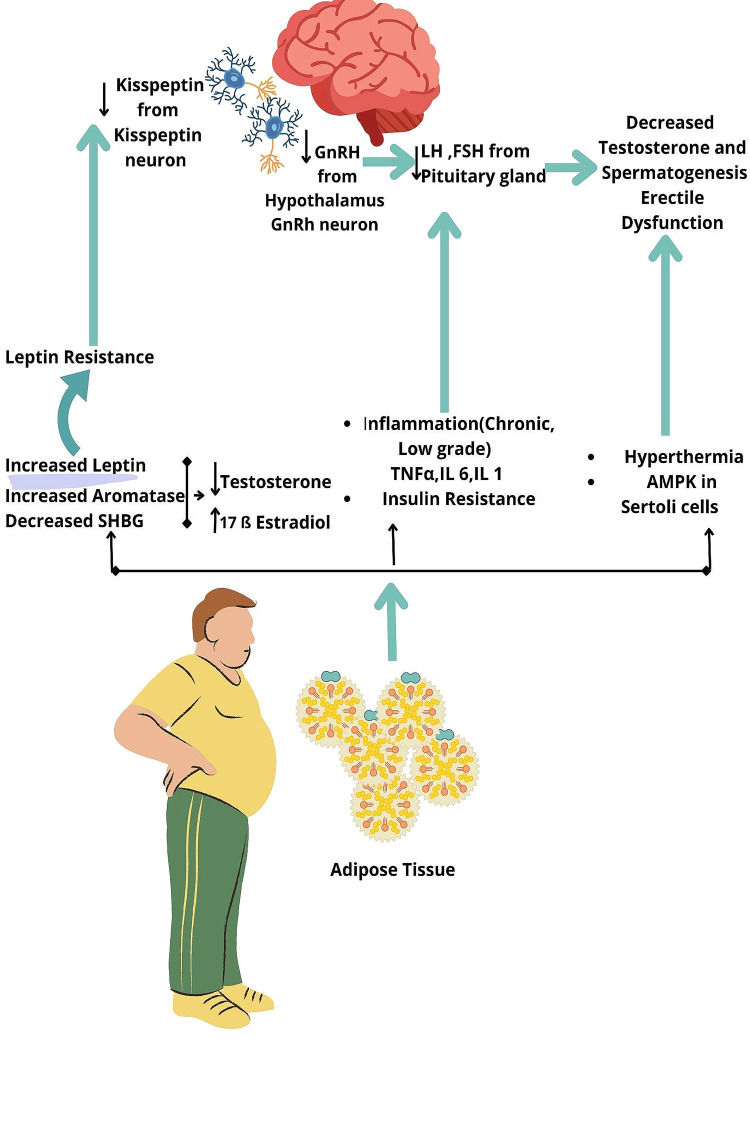
Hormones, cytokines, and adipokines acting at the level of brain and gonads in obesity resulting in decreased levels of testosterone, spermatogenesis, and erectile dysfunction. Obesity causes releases of leptin, increases aromatase activity, and decreases sex hormone binding globulin with decreases in testosterone and increases in estrogen levels; leptin resistance in obesity decreases kisspeptin and, in turn, decreases gonadotropin-releasing hormone from hypothalamus and decreases LH and FSH from pituitary gland; insulin resistance, TNFα, IL in chronic inflammation; hyperthermia, AMPK in Sertoli cells. SHBG: sex hormone binding globulin. AMPK: AMP-activated protein kinase. LH: luteinizing hormone. FSH: follicle stimulating hormone. TNFα: tumor necrosis factor alpha. ↓: Decrease. ↑: Increase. Image Credit: Rahnuma Ahmad.

There are several molecular mechanisms that affect male reproduction due to obesity (Figure 3). These include hypogonadism with its influence on spermatogenesis, oxidative stress, and inflammation [141-143]. The parameters of sperm, both bio-functional and conventional, are negatively affected by obesity. Inflammation in the microenvironment and adipocyte dysregulation affects insulin signaling, which may cause deterioration of the function of the testes [9,144,145].

**Figure 4:**
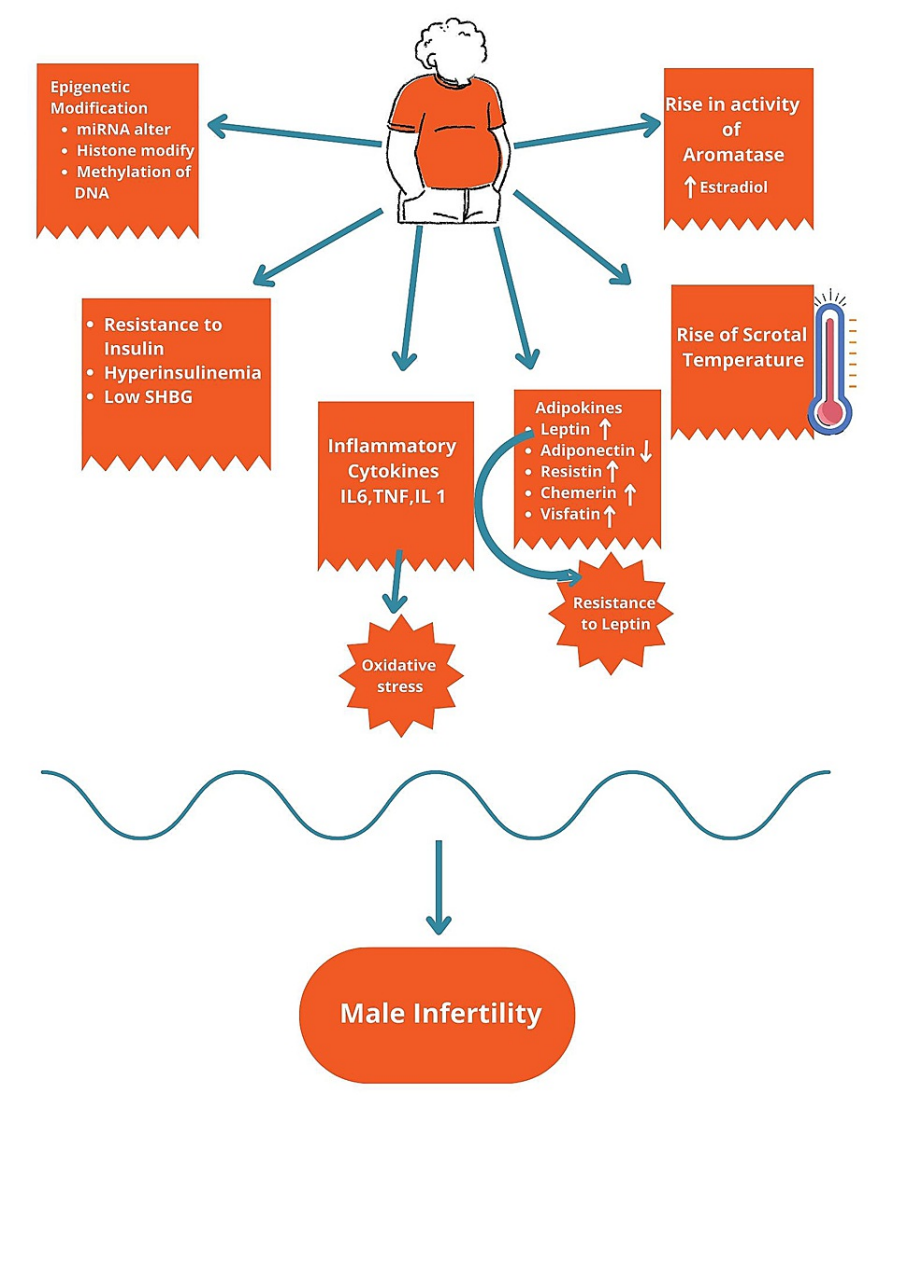
The complications of obesity in males leading to infertility, including increased aromatase activity and rise in estrogen level; increase in scrotal temperature; adipokines like leptin, chemerin, resistin, visfatin increase and decrease in adiponectin; leptin resistance; release of inflammatory cytokines and oxidative stress; insulin resistance, hyperinsulinemia and decrease in sex hormone binding globulin; epigenetic modification. SHBG: sex hormone binding globulin. TNF: tumor necrosis factor. ↓: Decrease. ↑: Increase. Image Credit: Rahnuma Ahmad.

Abnormal parameters of sperm in obese and overweight male subjects have been observed in several studies [14-16]. Mushtaq et al. in 2018 noted a statistically significant reduction associated with rising BMI in the case of men and a decrease in live birth and pregnancy rate with OR: 0.88, 95%CI 0.82-0.95, p=0.001 and OR: 0.78, 95%CI 0.63-0.98, p=0.03, respectively [17]. Campbell et al., in their meta-analysis, observed a significantly higher infertility risk among couples where the male partner was obese compared to couples with male partners having normal weight with OR: 1.66, 95%CI 1.53-1.79. Also, ART success was negatively impacted by male obesity [14]. Male obesity may be an equal contributor to infertility pathogenesis and embryo quality, as is obesity in females [18]. Figure 4 depicts the possible effects of obesity on male fertility.

Obesity and changes in hormones in male

Hypothalamic-pituitary-gonad axis controls the production of testosterone. LH and FSH are released from the anterior pituitary gland as a result of pulsatile GnRH hormone secretion [146]. Testosterone, if secreted from the testes due to the action of LH on Leydig cells and FSH, produces its effect on Sertoli cells, promoting sperm production [147]. Impairment of male sex hormones has been noted in obese men compared to those with normal body weight [148]. There is a reduction in sex hormone binding globulin, inhibin B, and free as well as total testosterone levels in the serum as a result of excess visceral fat. A rise in aromatase activity in obese individuals causes a surge in testosterone conversion to 17-β-estradiol, thus lowering the testosterone to 17-β-estradiol ratio [149,150]. A vicious cycle ensues as the activity of aromatase raises fat mass in the body with a rise in fat accumulation [147].

An increase in estrogen levels causes inhibition of the hypothalamic-pituitary-gonad axis with suppression of kisspeptin neurons, decreasing testosterone production [151]. Estrogen also inhibits both Leydig and Sertoli cells and thus suppresses testosterone secretion and sperm production [147]. Also, inflammatory cytokines and adipokines production are stimulated by adipose tissue and lead to decreased testosterone formation [9,152].

Male fertility and insulin resistance in obese individuals

Insulin signaling in tissues sensitive to insulin is affected significantly by high levels of inflammatory cytokines and adipokines and aggravates hyperinsulinemia and insulin resistance. Insulin has been noted to stimulate the hypothalamic-pituitary-gonad axis in cell culture studies [153]. In obese subjects, reduced serum sex hormone binding globulin due to hyperinsulinemia may result in a higher biological effect of estrogen [154]. Hyperinsulinemia also causes damage to mitochondrial and nuclear DNA and thus suppresses spermatogenesis [155,156]. The negative impact of hyperinsulinemia on androgens and testicular function results in hypogonadism in obese males [156,157]. Since an interrelationship exists between dysfunction of visceral fat, malfunction of testes, and insulin resistance, hypogonadism worsens insulin sensitivity, promotes the proliferation of adipocytes and body fat, and therefore enters a vicious cycle of metabolic syndrome affecting sperm quality and causing infertility [158,159].

Parameters of sperms in obesity

A study noted teratozoospermia and asthenozoospermia, both abnormal parameters of sperm in the case of obese and overweight men [160]. A study by Chavarro et al. observed that lower sperm count was associated with a BMI of more than 25 kg/m^2^ [149]. Another meta-analysis carried out in 2013 found a higher prevalence of oligospermia and azoospermia in obese and overweight men [161].

Changes in hormone levels may result in the impairment of spermatogenesis in the case of obese individuals [6]. Also, the temperature within the scrotum may increase as a result of the adipose cells in the region above the pubis and surrounding pampiniform plexus. Thus, the concentration and motility of sperm may be reduced, and DNA of sperm fragmentation and a rise in oxidative stress may occur [162].

Oxidative stress, parameters of sperm in obesity

A study on sperm parameters reported a higher percentage of spermatozoa having reduced membrane potential in mitochondria, DNA fragmentation, and release of phosphatidylserine. All these changes are considered apoptosis early markers [160]. Mitochondria provide sperm energy for motility and fertilization, employing oxidative phosphorylation and glycolysis. Since mitochondria produce oxidative agents, oxidative imbalance occurs when there is mitochondrial dysfunction which therefore hampers the function of sperm [163,164].

The study by Wang et al. noted high production of ROS and low membrane potential of mitochondria in individuals with infertility [165]. Another study reported the association between the low membrane potential of mitochondria in sperm and raised spermatozoa rate with the compactness of chromatin that is abnormal and indicated that damage to mitochondria might cause the DNA of sperm to be altered [166]. Kort et al. observed significantly raised DNA fragmentation in sperm in the case of obese subjects when compared to men with normal weight [167]. Another study found a higher spermatozoon with DNA fragmentation count in obese individuals [149].

Obesity is linked to an increase in the formation of inflammatory cytokines like TNF-α and IL-6, which results in chronic inflammation [168]. ROS at physiological levels promote acrosomal reaction and capacitation, but at higher levels, they cause oxidation and damage DNA, lipid, and protein [169]. Lack of balance between the formation of oxidants and antioxidation activity present within the seminal fluid in humans. ROS may oxidize the double bonds in the membrane lipids of spermatozoa composed of polyunsaturated fatty acids. This outcome is the peroxidation of lipids and lower fluidity of the membrane. The ROS may also damage the DNA of sperm, and since there is the absence of systems of enzymes in the cytoplasm needed for the repair of DNA at the molecular level, DNA repair in spermatozoa is not possible [170-172].

The proteome of seminal plasma and obesity

Proteomics is the protein analysis of biological fluid, tissue, and cell [173]. The pattern of expression of proteins within the seminal fluid in individuals with conditions like obesity resulting in high oxidative stress has been studied in recent times [174]. The proteomes in the seminal fluid may act as oxidative stress biomarkers for spermatozoa [175]. Overexpression of certain proteins responsible for oxidative stress, like S100A9 (protein S100 calcium-binding protein) and haptoglobin within the seminal fluid of individuals suffering from obesity, have been noted in a recent study conducted by Herwig et al. [176]. S100A9 can induce a cascade of inflammation through binding with inflammatory receptors and haptoglobin, which has antioxidant properties. Other antioxidants that increase in obese individuals' seminal plasma include clusterin, ceruloplasmin, adenosine diphosphate ribosyl cyclase, and glutathione peroxidase to create a balance between the system of oxidant and antioxidant so that ROS-induced damage to cells may be minimized [176,177].

Reproduction in males, obesity, and adipokines

Adipokine is a molecule formed by adipose tissues, which, under a physiological state, participate in body homeostasis maintenance and the modulation of immune system activity [178]. On the other hand, excess production of adipokine in the case of obesity results in a chronic inflammatory state. Macrophages in obese individuals are stimulated by non-esterified fatty acids released by adipocytes to produce large quantities of TNF-α. The TNF-α then further promoted the production of non-esterified fatty acids, cytokines like IL 1β, L-6, chemokines, and acute phase protein from adipocytes which cause chemoattraction of leukocytes like monocyte and macrophage to adipocytes [179-181]. Thus there ensues a vicious cycle that ultimately causes chronic systemic inflammation [182]. Adipokine studied for male fertility is leptin, elaborated in the following section.

Obesity, leptin, and male infertility

Leptin is an adipokine produced by adipocytes and encoded by the obesity gene, and its level is associated positively with the size of adipocytes and body fat percentage [9]. Leptin is a satiety hormone since it lowers food intake and suppresses appetite. This occurs as it represses the neuropeptide Y encoding gene and induces amphetamine-regulated transcript and proopiomelanocortin encoding gene by binding to leptin receptors in the hypothalamus. Along with its food intake regulating role, it plays a significant part (both centrally and peripherally) in reproduction [183]. Studies have observed LH secretion is stimulated by leptin [184]. Leptin’s central role in reproduction is mediated through kisspeptin since leptin receptors are not found on GnRH neurons. Kisspeptin receptors are found in GnRH neurons and stimulate GnRH, which in turn causes the secretion of FSH and LH [185,186].

Signaling pathways activated by leptin include a signal transducer and activator of transcription protein (STAT-5 and STAT-3), Janus kinase, phosphoinositide 3 kinase, extracellular signal-regulated kinase/mitogen-activated protein kinase (MAPK), nitric oxide and mammalian target of rapamycin (mTOR). However, higher levels of adipokine negatively impact fertility in males. Excess quantities of leptin secreted in obesity by adipocytes lead to the hypothalamic-pituitary axis becoming leptin signal resistant, possibly due to overstimulation of negative feedback regulators like T cell protein tyrosine phosphatase, protein tyrosine phosphatase 1B, and suppressor of cytokine signaling. These regulators have been raised in obese animals’ hypothalamus [57,187]. A high-fat diet in animals has been shown to result in resistance to leptin and a fall in the expression of kisspeptin in the arcuate nucleus and third ventricular rostral periventricular area [188]. Thus central hypogonadism occurs since the decrease in kisspeptin inhibits GnRH neurons lowering gonadotropin and secretion of testosterone [9,189].

A positive association has been observed between BMI, leptin level in serum, and sperm parameters alteration [190]. A case-control study in humans noted raised level of leptin, a decrease in the concentration of sperm, and high sperm DNA fragmentation in obese men compared to men with normal weight [191]. Leptin raises species of reactive oxygen and fragmentation of sperm DNA as observed in an animal study [192]. In the male reproductive system, leptin binds to its receptors, thus activating the PI3K pathway within the testes, raising oxidative stress, and causing disruption of the transition of histone to protamine. This leaves the sperm DNA exposed to free radical attacks, which may lead to sperm DNA fragmentation and apoptosis [183].

Studies in rat Leydig cells have noted that high leptin, similar to that found in obesity, suppresses testosterone secretion [193,194]. There may be AMPK pathway up-regulation in case of high leptin levels, hindering steroidogenic acute regulatory protein and cytochrome P450 family 11 subfamilies A member 1 in Leydig cells. There may also be STAT transcriptional activity downregulation and a decrease in the cAMP-dependent steroidogenic gene, thus hampering testosterone formation [9,195]. High levels of leptin also inhibit Sertoli cells' nutrition by lowering the production of acetate from glucose [9]. Therefore, high leptin level in obesity impacts testosterone formation in Leydig cells and negatively affects Sertoli cells, thus altering testicular immune defense affects the blood-testis barrier hampering spermatogenesis [188]. Mitochondrial activity is modulated by leptin. High leptin levels cause oxidative stress by altering mitochondria function and adversely affecting sperm [196].

Obesity, adiponectin, and male infertility

Adiponectin is an adipokine that, unlike most other adipokines, has anti-inflammatory activity with a negative association with fat mass in the body. It has a structure similar to complement C1q, collagen IV, VIII, and TNF-α [178]. This adipokine may have a central effect since its receptors are found in the pituitary gland [182]. Studies on animals have revealed that adiponectin has receptors in Leydig cells, germ cells, and Sertoli cells. This adipokine may directly exert its action on the testes regulating spermatogenesis and steroidogenesis [182,197].

The adiponectin level in the seminal fluid has been associated positively with the total count of sperm, concentration of sperm, and normal morphology of mature sperm [197,198]. Protection against the detrimental consequences of cytokines released due to inflammation like IL 1β and TNF-α on Leydig cells may be provided by adiponectin [190]. This adipokine promotes phosphorylation of AMP-dependent protein kinase and causes inhibition of translocation in the nucleus of nuclear factor β [199,200]. Adiponectin also promotes insulin sensitivity [201]. Adiponectin concentration in the seminal fluid was found less in subjects who were obese and overweight in comparison to individuals having normal weight [198,202].

Obesity, resistin, chemerin, visfatin, and male infertility

Resistin promotes insulin resistance and has been associated positively with markers of inflammation [178,203]. Resistin has been found in seminiferous tubules and Leydig cells in rats [198]. A few studies have been carried out on resistin in seminal fluid. One such study observed a negative association between the level of resistin in seminal fluid and sperm vitality and motility, as well as a positive association with markers of inflammation in semen like elastase and IL-6 [204]. This suggests a vital role of resistin in seminal fluid and male reproductive system inflammation [182]. The role of visfatin is unclear in obesity since certain studies have observed high concentrations of visfatin provoke insulin resistance, while other research has noted its protective role [205,206]. Visfatin has been located in spermatozoa, spermatocytes, and Leydig cells [197].

Chemerin (a recently found adipokine that modifies the action of insulin) has been significantly raised in obese and overweight subjects compared to subjects with normal weight and showed a positive association with rising BMI [207,208]. Its receptors have been located in the Leydig cells of both rats and humans. Chemerin has exhibited a suppressive effect on sperm production [209,210]. Chemerin suppresses the synthesis of testosterone in the Leydig cell culture. In humans, a negative association was noted between the concentration of chemerin and the motility of sperm [208].

Epigenetic modification in obesity

Children's health (both reproductive and metabolic) is impacted negatively by paternal obesity. Several studies have observed that those born to obese male parents have a greater chance of becoming obese [9,211]. The gene expression can be modified by environmental factors and the processes that change the gene's activity, including alteration of chromatin structure without modification of nucleotide sequence, known as epigenetics [212-214].

In the case of the male, methylation of DNA is imperative for spermatogenesis. For X chromosome inactivation during meiotic cell division and paternal gene imprinting in sperm, sperm methylation is essential [215,216]. Infertility and pregnancy loss have been observed in cases of abnormal sperm methylation [216]. DNA methylation change has been noted in obese and overweight individuals’ spermatozoa compared to men with normal weight in the imprinted gene regulatory region [217]. Maternally expressed gene 3 (MEG3), epsilon sarcoglycan (SGCE)/paternally expressed gene 10 (PEG10), small nuclear ribonucleoprotein polypeptide N (SNRPN), and necdin (NDN) are some of the epigenetically modified genes in obese male individuals. These modified genes play a role in fetus development and tumor growth [218].

**Figure 5:**
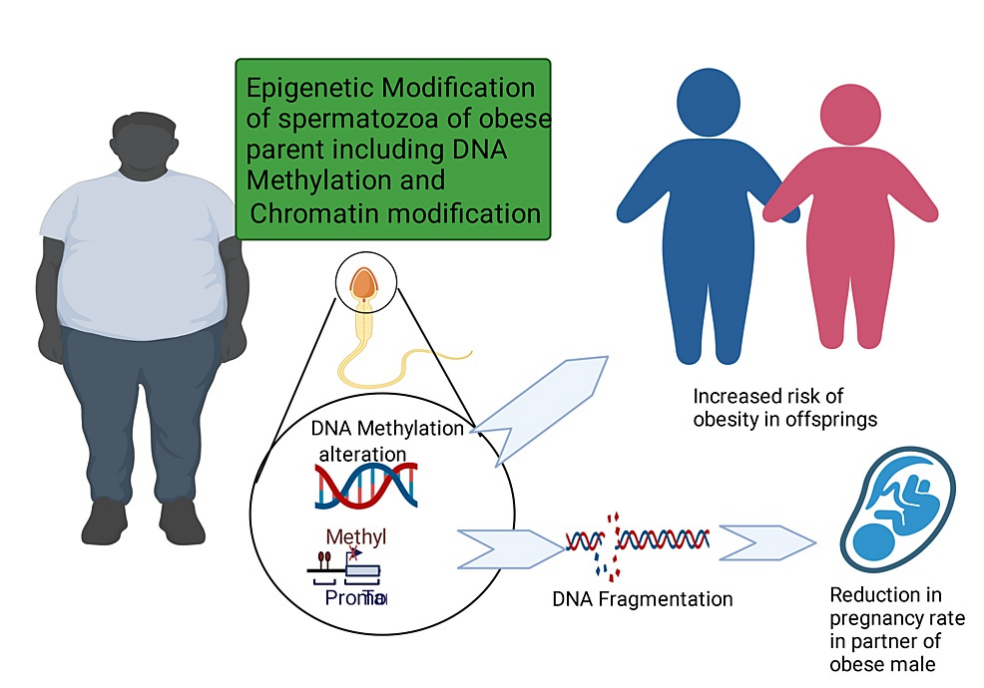
The impact of obesity on sperm DNA, leading to reduced pregnancy rate in partners of obese males and also an increase in obesity risk in the offspring of obese male individuals. This figure has been developed using BioRender (https://biorender.com/) License number: FR24I07PEF. Image Credit: Rahnuma Ahmad.

It was reported in a study that the difference in methylation of DNA between normal and obese male subjects was significant while mapping the epigenetic pattern in sperm of both normal and obese men [219]. Altered methylation of imprint gene was linked to raised fragmentation of sperm DNA and reduced rate of pregnancy [220,221]. Also, changed methylation of DNA in several imprint gene regulatory regions has been observed in children born to obese male parents [218,222]. Studies also noted that losing weight may reverse the epigenetic alterations related to obesity, thus lowering offspring's adverse effects [223]. Figure 5 depicts the impact of obesity on sperm DNA, leading to a reduced pregnancy rate in partners of obese males and also an increase in obesity risk in the offspring of obese male individuals.

The outcome of assisted reproductive technology in obese males

There are several studies that suggest that male BMI above the normal range may be related to a reduced rate of success following the use of ART like IVF and intracytoplasmic sperm injection (ICSI) [12,224,225]. A study performed by Anifandis et al. showed that the effect of paternal BMI on the quality of the embryo and outcome of IVF was greater than the parameters of semen analysis [226]. Zhao et al. found that damage to sperm resulting from male obesity was associated with a higher rate of miscarriage and a lower rate of pregnancy in ICSI and IVF cycles [227]. A retrospective study was done to assess the pregnancy rate in 290 cycles of IVF and ICSI. The study found that a male BMI greater than 25.0 kg/m² affected the pregnancy rate negatively following IVF. The likelihood of clinical pregnancy per IVF cycle was reduced by 79% for men having BMI above 25 kg/m^2^ [228]. Another retrospective study done to analyze the 305 IVF-ICSI cycles found high male BMI was related to poorer development of blastocyst and decreased clinical pregnancy rate and live birth rate. Statistically significant linear reduction in pregnancy rate with increasing paternal BMI from normal to obese men (p<0.01) was noted [229]. The study by Mehri et al. included 344 IVF-ICSI cycles, and the researchers concluded that male BMI above normal has a negative impact on pregnancy rate following IVF-ICSI cycles [230]. A lower pregnancy rate, live birth, and implantation rate was associated with raised male BMI in another retrospective study, which included 177 ICSI cycles [231]. However, Schliep et al. and Thomsen et al. found no significant association between raised male BMI and IVF-ICSI outcome [232,233].

Lifestyle, obesity, and infertility

A sedentary lifestyle and unhealthy eating habits have significantly contributed to obesity prevalence globally. An increase in the circumference of the waist by 3.1 cm has been reported when there is increased sedentary time by 10% [234]. Also, it was noted in a study that weight gain was due to sedentary time [235]. The general well-being of an individual is enhanced when one performs exercise regularly, which also protects against obesity to a certain extent. There is an increase in sensitivity to insulin, which promotes the function of ovaries and enhances the probability of conception [236]. Metasets et al. found in their randomized control trial that the obese/overweight subjects who underwent diet and exercise intervention programs for six months before taking part in 18 months of fertility treatment had significantly higher rates of conception than the control group [237]. Another cohort study done by Wise et al. observed physical exercise to be positively related to fecundability in the case of obese and overweight women [238]. Such findings suggest that a sedentary lifestyle is a modifiable risk factor for infertility [236].

Nutritional factors like a high-fat diet have been observed to suppress reproduction in male subjects by influencing the molecular and physical structure of cells of sperm, fetus, and offspring [239]. A reduction in the diameter and height of seminiferous tubules and seminiferous epithelium has been reported in high-fat diet-fed mice [240]. On the other hand, consumption of a diet rich in fish, legumes, vegetables, and fruits can improve the quality of sperm and decrease in fragmentation of DNA compared to those who do not have such a diet [241]. A study performed in 2016 on infertile women suffering from obesity found that lifestyle intervention (minimization of environmental toxicants, systemic disease, smoking, alcohol, and socioeconomic status that contribute to obesity) improves reproductive health and results in a rise in the rate of natural conception and advised maintenance of healthy dietary habits to avoid an accumulation of body fat which hampers ovulation [242]. Bariatric surgery is an option for weight loss in obese men, it may normalize the hormone profile but will not improve semen parameters until two years post-surgery. Slow/milder weight loss is associated with improved sperm function in obese men [243-245]. Thus, in order to improve fertility among obese and overweight individuals, lifestyle habit evaluation and unhealthy habit alteration through management imparted by trained healthcare providers are important [246].

Limitations of the study

The study faced certain limitations: I. Since it is a narration review, no meta-analysis was done II. Excluded from the study was research work not available in the English language. III. Articles that require to be purchased to be accessed could not be included in the study. Additionally, this paper focuses mainly on obesity-induced factors leading to infertility. We had not included therapeutic options in detail in this narrative review.

## Conclusions

As the obesity epidemic continues to grow, more individuals (both women and men) are likely to suffer complications in both metabolic and reproductive health. Several hormones, growth factors, cytokines, and adipokines acting at the level of the brain and gonads link obesity to reproductive dysfunction. Since obesity is a state of chronic inflammation, in women there may be raised macrophage infiltration in ovaries by pathways mediated through MCP-1. Raised serum AGEs may also heighten dysfunction of ovaries linked to adiposity. Obesity-associated male infertility includes several molecular pathways, and disturbance is created by obesity in hormonal balance and parameters of sperm.

To raise our ability to find means for preventing and managing obesity and infertility, it is necessary to understand the molecular mechanisms connecting obesity and fertility impairment and obesity. Each of the involved molecules and the possible pathways provide a therapeutic target so that the reproductive health of obese/overweight individuals may be improved. It is imperative to discover the molecular mechanisms which impact almost all levels of the hypothalamic-pituitary-gonadal axis. Thus, a more extensive study must be performed to comprehend the molecular mechanisms linking obesity to infertility. Since shedding extra weight is often a challenge that is unsustainable, developing therapeutics for reproductive dysfunction for the betterment of reproductive health in obese subjects is necessary. Also, awareness in regard to the harmful impact that obesity poses on reproduction and a healthy lifestyle needs to be encouraged. Governments must work with healthcare providers to create awareness and policies to prevent and manage obesity. It is a matter of concern since not only does obesity causes infertility, but the epigenetic modification in obese males may also be transferred to their offspring.
